# A New Genotype of *Trichophyton quinckeanum* with Point Mutations in *Erg11A* Encoding Sterol 14-α Demethylase Exhibits Increased Itraconazole Resistance

**DOI:** 10.3390/jof9101006

**Published:** 2023-10-12

**Authors:** Paula Winter, Anke Burmester, Jörg Tittelbach, Cornelia Wiegand

**Affiliations:** Department of Dermatology, Jena University Hospital, Friedrich Schiller University, D-07747 Jena, Germany

**Keywords:** *Trichophyton quinckeanum*, *Trichophyton schoenleinii*, *Erg11A*, *Erg11B*, sterol 14-α demethylase, Erg1, squalene epoxidase, mating type

## Abstract

*Trichophyton quinckeanum*, the causative agent of mouse favus, has been responsible for several infections of animal owners in recent years and showed an infection peak around 2020 in Jena, Thuringia. The isolated *T. quinckeanum* strains from Thuringia differ in some positions of the ITS region compared to strains from the IHEM collection as well as to *Trichophyton schoenleinii*. All *T. quinckeanum* strains of the new genotype show up to a 100-fold increased itraconazole resistance as measured by microplate laser nephelometry (MLN) assays. Analysis of genes involved in *Trichophyton indotineae* azole resistance, such as *Erg1*, which encodes squalene epoxidase, and *Erg11B*, one of two copies of the sterol 14-α demethylase gene, show a 100% identity between the two *T. quinckeanum* genotypes. In contrast, *Erg11A* fragments differ in 15-nucleotide positions between both *T. quinckeanum* genotypes, resulting in the unique amino acid substitution Ala256Ser in resistant strains. The new *T. quinckeanum* genotype may have evolved through interspecies mating. Mating type analysis showed a nearly 100% identity of the minus type *MAT1-1-1* fragment for all *T. quinckeanum* isolates. The closely related *Trichophyton schoenleinii* belongs to the plus mating type and has 100% identical fragments of *Erg1* and *Erg11B*. Erg11A protein sequences of *T. schoenleinii* and *T. quinckeanum* showed increased diversity.

## 1. Introduction

*Trichophyton quinckeanum* is the causative agent of favus in mice. H. I. Quincke, who was honored as the species’ namesake, presumed that several fungi could cause the clinical symptoms of favus in humans [1]. Favus is a condition that most commonly affects the scalp in humans, where fungal hyphae invade the skin around hair follicles and form a cup-shaped, yellow scab. Quincke’s excellent drawings of microscopic analyses of the described ‘α-fungus’, which he found in human favus, show typical micro- and macroconidia [1]. He also infected animals such as mice with the ‘α-fungus’ and was able to successfully trigger the disease in these mice with the typical favus symptoms [1]. Today, *Trichophyton schoenleinii* is the causative agent of most cases of favus in humans [2], which was first described by J. L. Schoenlein and R. Remak [2] and corresponds to the ‘γ-fungus’ description of H. I. Quincke [1]. Nevertheless, rare cases of human favus due to *T. quinckeanum* have sometimes been identified [3]. Phylogenetic analyses showed that both species are closely related [4]; however, the morphology of *T. quinckeanum* and *T. schoenleinii* differs significantly; the latter is incapable of producing micro- or macroconidia and the mycelium displays chandelier- or antler-like hyphae [2].

Favus in humans has been gradually disappearing in Europe and is more prevalent in Middle East and Africa, although the trend is also declining [2]. However, resurgence of favus in humans is possible [5], particularly if people do not have access to or avoid medical treatment. Interestingly, the number of patients presenting with *T. quinckeanum* infections has increased significantly in recent years in the outpatient clinic of the Department of Dermatology at Jena University Hospital [6]. Here, infections caused typically tinea corporis or tinea capitis [6]. Severe tinea capitis due to the new *T. quinckeanum* subtype has been mainly observed in young children, but not in adults or adolescents, where tinea corporis was the typical diagnosis [6]. Several of the infected individuals were cat owners [6]. Two of the investigated *T. quinckeanum* fungal cultures were isolated from animal hair of the pet owners’ cats. The infection peak in 2020 correlated with a peak in the field mouse population (*Microtus arvalis*) in agriculture in the Jena area, according to reports from the Thuringia ministry of agriculture (TLLLR) [7].

Genetic analysis of the *T. quinckeanum* strains isolated in Jena showed that they differ in few positions in the ITS region compared to the neotype strain IHEM 13697 [8]. Strains with this new ITS pattern were also observed in other regions of Germany [9,10]. Recently, *T. quinckeanum* strains of this new ITS genotype were isolated in the Czech Republic [11]. First isolates were detected in 2016, and increased numbers of strains were reported up to 2020. Cats and dogs often transmit the disease to their owners [11]. A recent Swiss study showed that cats with dermatomycosis are more likely to be infected with *T. quinckeanum* when kept outdoors and thus have the opportunity to hunt mice [12]. The comparison of epidemiological studies is often hampered by name changes and reclassifications of dermatophytes. For example, the reference strain CBS 318.56 of the Swiss study mentioned was first classified as *T. mentagrophytes* [12] and later reclassified as *T. quinckeanum* [8].

In recent decades, changes in species designation based on morphological and molecular datasets have become more frequent. The species identification debate surrounding *T. quinckeanum* started with analysis of mating behavior [13,14]. In the late 1990s, the species name was changed into *T. mentagrophytes* due to the mating behavior [15,16]. However, it was then renamed to *T. quinckeanum* in 2017 [8]. Weitzman and Padhye demonstrated the ability of *T. quinckeanum* to produce viable ascospore progeny with both *T. simii* isolate and *T. benhamiae* [14]. Interestingly, all *T. quinckeanum* isolates of this study belonged to the minus mating type [14]. The combination of phylogenetic data based on a range of DNA sequences from multiple genes as well as morphological and physiological data sets allows the identification of new species or subtypes. An example of this is *T. benhamiae var. luteum*, classified according to the new classification system [17] and formerly referred to as the yellow subtype of *T. benhamiae* [18]. Another example of a recently identified new species is *T. indotineae* [19,20], a novel human pathogen that exhibits multiple drug resistances to allylamines and azoles [21,22,23]. How these new genotype species evolved when clonal reproduction appears to be the main mode of reproduction in *Trichophyton* species is an open question and part of the current debate [24]. So far, Metin and Heitman have described clonal reproduction of anthropophilic species like *T. rubrum* or *T. interdigitale*, while sexual reproduction is believed to be restricted to zoophilic strains such as *T. mentagrophytes* or *T. benhamiae* [24]. Hence, it is important to elucidate how the new subtype of *T. quinckeanum* evolved and why we did not recover its old subtype in our patients.

The objective of this study was to investigate the sensitivity of the collected *T. quinckeanum* strains from Thuringia that show differences in the ITS sequence compared to the IHEM strains as well as *Trichophyton schoenleinii* towards common antifungal azoles. Additionally, analysis of genes involved in azole resistance, such as *Erg1*, *Erg11A* and *Erg11B*, was performed to identify point mutations responsible for amino acid substitutions, which could account for the sensitivity differences to azoles. Finally, yet importantly, mating type analysis was carried out for the *T. quinckeanum* isolates to determine whether the new genotype may have evolved through interspecies mating.

## 2. Materials and Methods

### 2.1. Strains and Growth Conditions

*T. quinckeanum* strains from the Jena University Hospital (UKJ) were stored as glycerol stock solutions at −80 °C and cultured on dermasel agar and Sabouraud Dextrose (2% *w*/*v* Dextrose, SDA) agar plates. For microscopy, strains were cultivated on Takashio agar [25,26]. IHEM strains were purchased from the Belgian Coordinated Collection of Microorganisms BCCM/IHEM (Brussels, Belgium). Strain ATCC 46950, originally classified as *T. schoenleinii* [27], was obtained from the American Type Culture Collection ATCC (Manassas, VA, USA). *T. schoenleinii* UKJ 1317/12 was a gift of H.-J. Tietz, head of the Mycoclinic Berlin. Source information on the isolates is provided in Table 1.

### 2.2. Plate Assay for Resistance Estimation of T. schoenleinii and T. quinckeanum

*T. schoenleinii* does not form micro- or macroconidia; therefore, pieces of mycelium were transferred to liquid or solid media. Methods based on optical density measurements were not suitable because large particles lead to an inhomogeneous solution, which influences the measurements results. An agar-based resistance assay was developed for *Aspergillus fumigatus* [30] and was adapted to estimate resistance pattern of *T. schoenleinii*. Small plates were filled with an 8 mL SDA agar. One plate was used as a control without antifungal compounds, and three further plates contained increasing concentrations of fluconazole (0.4 µg/mL; 4 µg/mL; 40 µg/mL) or itraconazole (0.005 µg/mL; 0.05 µg/mL; 0.5 µg/mL). Concentration ranges were estimated from the results of *T. quinckeanum*. The plates were inoculated with small pieces (around 0.5 × 1 cm^2^) of *T. schoenleinii* or *T. quinckeanum* mycelium obtained from pre-cultivated plates grown for three to eight weeks. The pieces were excised from the growth front zone. Growth patterns were assessed after 5 to 7 days and compared to growth on SDA control plates without antifungal agents. Positive results were obtained when mycelium was able to grow from the transferred agar piece on antifungal-containing agar plates similar to the control plate without the addition of antifungal compounds. Negative results were obtained if the mycelial growth was restricted to the transferred agar piece. An intermediate reaction was classified as growth within the restrictive agar plate being distinctly reduced in comparison to the control.

### 2.3. Microplate Laser Nephelometry Assays

The MLN assays allow the recording of growth curves in each single well of a 96-well microplate. The analyses were performed as previously described [23,31,32] over 120 h at 30 °C with continuous orbital shaking and hourly measurements employing the NEPHELOstar Galaxy (BMG Labtech, Ortenberg, Germany). Spores were collected from pre-cultivated SDA or Dermasel agar plates pre-cultivated between three and eight weeks at room temperature. The spores were suspended in a 5 mL sterile, isotonic NaCl solution (9 g/L; Fresenius Kabi, Bad Homburg, Germany) and the suspension was then filtered through a cell strainer with a mesh size of 40 µm (Greiner Bio-One, Frickenhausen, Germany) to remove mycelial debris. Spore concentration was estimated by counting spores using disposable counting chambers (type Neubauer improved; Carl Roth GmbH, Karlsruhe, Germany), and suspensions were adjusted to a final concentration of 2 × 10^3^ spores/mL. A total of 100 µL of the adjusted spore solution was added to each well, which resulted in approximately 200 spores per well. Adjusted spore solutions were also plated on SDA agar to evaluate spore viability and allow determination of numbers of colony-forming units. The antifungal compounds itraconazole, voriconazole, sertaconazole-nitrate, terbinafin and nystatin were solved in DMSO and stored as stocks as described [23]. Ciclopirox olamine was prepared as previously reported [32]. Amorolfin (EP standard, Sigma Aldrich GmbH, Taufkirchen, Germany) was stored as a stock solution of 1 mg/mL in DMSO and clotrimazole (PHR Standard, Sigma Aldrich) was stored as a stock of 5 mg/mL in DSMO. Antifungal stocks were diluted in a liquid SDA medium in 1:2 increments, reducing the concentration by half in each case. As positive control SDA media without antifungals (100 µL per well) was added to the spore solution, blanks of each type were included as negative controls with the spore solution being replaced by the SDA medium. For each strain, three technical and two biological replicates were obtained. The concentration of a 90% growth inhibition (MIC_90_) of the antifungals was calculated from the growth curves over 120 h as described previously [23]. The following antifungal concentration ranges were used: itraconazole (1 µg/mL to 0.0005 µg/mL), voriconazole (2 µg/mL to 0.008 µg/mL), sertaconazole-nitrate (8 µg/mL to 0.13 µg/mL), clotrimazole (1 µg/mL to 0.016 µg/mL), terbinafin (0.05 µg/mL to 0.0008 µg/mL), amorolfin (0.13 µg/mL to 0.002 µg/mL), nystatin (40 µg/mL to 0.63 µg/mL) and ciclopirox olamine (32 µg/mL to 0.5 µg/mL).

### 2.4. DNA Isolation and DNA Amplification

*T. schoenleinii* and ATCC strain 46950 DNA were isolated from fungal cultures grown in a SD broth in Erlenmeyer flasks under shaking (120 rpm) at room temperature to enhance the mycelial material. The mycelium was harvested using Miracloth (Merck Kg, Darmstadt, Germany) filter screens and treated with liquid nitrogen using a mortar and a pestle. *T. quinckeanum* DNA was obtained from cultures of at least two weeks of age on SD agar. Sufficient amounts of microconidia were formed within this period. For further DNA preparation, the Qiagen GmbH (Hilden, Germany) DNA mini kit was used according to the manufacturer’s instructions. Protease K treatment at 55 °C was limited to one hour for the liquid culture preparations but was performed overnight for mycelium forming microconidia. *Erg1*, *Erg11A* and *Erg11B* fragments were amplified as previously described [23]. ITS regions were sequenced to correctly identify all strains using primer V9G [33] and LSU266 [34]. The phylogenetic tree based on the Neighbor Joining method [35] (Figure S1) visualized the relationship of *T. quinckeanum* and *T. schoenleinii* genotypes. Mating type analysis was performed as described [26]. Primers specially designed for amplification of *T. quinckeanum* fragments are listed in Table S1. Sequence data were stored in GenBank; GenBank Acc No. OQ536505–OQ536519 for *Erg1* fragments; GenBank Acc. No. OQ536520–OQ536534 for *Erg11A*; GenBank Acc. No. OQ536535- OQ536549 for *Erg11B*, GenBank Acc. No. OQ536550–OQ536561, OQ536564 for the *MAT1-1-1* encoding α-box transcription factor and GenBank Acc. No. OQ536562, OQ536563 for the *MAT1-1-2* encoding HMG box transcription factor. ITS sequence data GenBank Acc. No. for *T. quinckeanum* were as previously described [6]. Strains and related GenBank Acc. No. are listed in Table S2.

### 2.5. Microscopy

Microscopy was performed with the Keyence digital microscope on mycelium-grown Takashio agar plates [25,26]. A piece of agar medium, about 0.5–1 cm wide and 3–4 cm long, was taken from of the petri dish and covered with sterile cellophane foil before starting fungal cultivation to improve the contrast for microscopic images. Microscopic images were obtained directly from grown culture of the plates without damaging the mycelium.

## 3. Results

### 3.1. Resistant Phenotypes of T. quinckeanum Strains

MLN was used to determine the 90% minimal inhibitory concentration (MIC_90_) for the antifungal agents (Table 2). Similar MIC_90_ values were found for most antifungals (Table 2). In contrast, an increase in itraconazole MIC_90_ values was observed when *T. quinckeanum* IHEM strains and UKJ collection strains were compared. IHEM and UKJ strains represent different *T. quinckeanum* ITS genotypes. All UKJ strains showed values in the range of 0.09–0.45 µg/mL for the MIC_90_ of itraconazole (Table 2) and represented one genotype. UKJ isolates 1953/19 and 1254/20 were isolated from cat hair provided by the infected pet owners. This showed how important it is that, in addition to treating pet owners, the infected pets should also be treated by a veterinarian. IHEM strains showed MIC_90_ values for itraconazole in the range of 0.003–0.07 µg/mL and represented a different genotype (Table 2). A comparison of the results for both *T. quinckeanum* genotypes exhibited an up to 100-fold increase in values for itraconazole (Table 2). Cut-off values have not yet been defined. Nevertheless, a cut-off of 0.08 µg/mL would separate both genotypes in sensitive and resistant strains. IHEM strain analysis allows a glimpse into the past, as these genotypes were isolated in different years and in different parts of the world. F. Blank of Philadelphia, USA isolated IHEM strain 13570 from a dog and strain 13697 from a mouse [4,14], respectively, named No. 19 (syn. CDC X395) and No. 10. (syn. CDC X393). Strain IHEM 13697 represents the neotype isolate of *T. quinckeanum* [8]. The IHEM 13572 strain was isolated from a rodent in Adelaide, Australia in 1964. Strain IHEM 26522 is a subculture of CBS 318.56, which causes deep trichophytosis in humans, and it was isolated in 1955 by J. Zoon in the Netherlands.

All *T. quinckeanum* strains demonstrated high resistance to fluconazole, and it was not possible to determine MIC_90_ values with the MLN assay. A fluconazole concentration of 160 µg/mL was not able to sufficiently inhibit the growth of *T. quinckeanum* strains. and the MLN assay failed.

**Figure 1:**
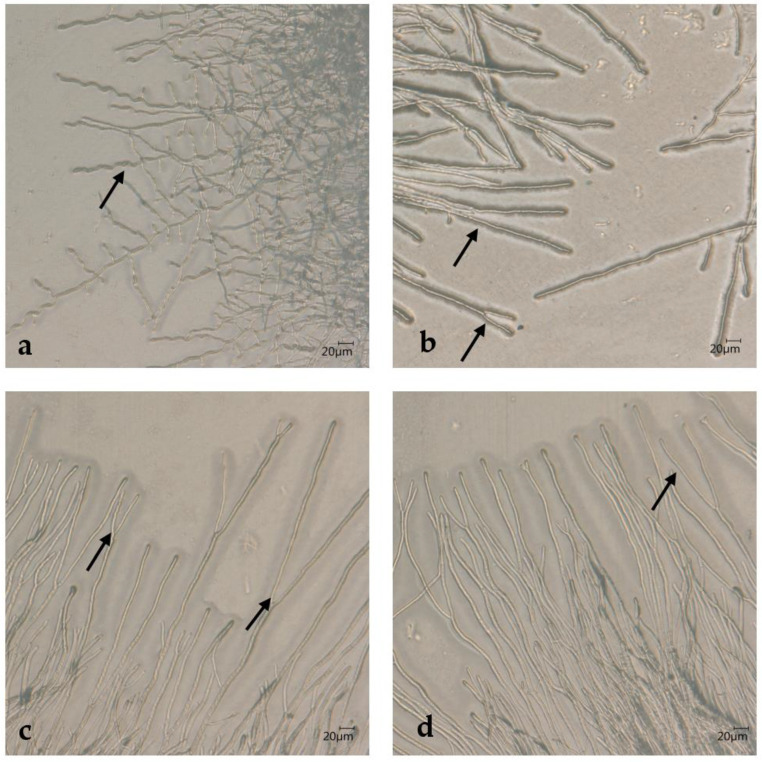
Morphology of *T. schoenleinii* strains IHEM 13515 (**a**) and UKJ 1317/12 (**b**) as well as *T. mentagrophytes* strain ATCC 46950 (**c**,**d**). ‘Antler’ hyphae show equal length of both hyphal branches and represent the main type of hyphae formed by UKJ 1317/12 and ATCC 46950 (**b**–**d**). Only a few examples of this type were visible for IHEM 13515 (**a**). Arrows mark typical ‘stag antler’ hyphae.

To understand the relationship between the two genotypes of *T. quinckeanum* and the close relative *T. schoenleinii*, two *T. schoenleinii* strains were also analyzed. IHEM strain 13515 was a human isolate from Casablanca, Morocco (obtained in 1966) [4] and UKJ strain 1317/12 was a gift of H.-J. Tietz isolated approximately 1972. ITS data confirm the species identification. Strain ATCC 46950 was isolated in Iraq from depilated human hair and identified as *T. schoenleinii* [36]. Genetic analysis showed that ATCC 46950 belongs to the *T. mentagrophytes* complex [4]. However, *T. schoenleinii* strains IHEM 13515 and UKJ 1317/12 as well as *T. mentagrophytes* strain ATCC 46950 form typical ‘stag antler’ hyphae as shown in Figure 1. Therefore, the morphology of ATCC 46950 closely resembles that of *T. schoenleinii*. ‘Antler’ hyphae are equal in length in both hyphal branches and often have an arc of about 45°. Interestingly, only a few hyphae of this type formed in IHEM 13515 (Figure 1a), while almost all hyphae observed in ATCC 46950 belonged to this type (Figure 1c,d). UKJ 1317/12 and ATCC 46950 showed high morphological similarities. Hyphae of *T. mentagrophytes* ATCC 46950 showed a coordinated growth front in large parts of the colony (Figure 1d).

Since *T. schoenleinii* and *T. mentagrophytes* strain ATCC 46950 do not form spores, an agar-based method was used for the analysis of azole resistance. The method is suitable to compare data of *T. schoenleinii* strains with *T. quinckeanum* strains, as shown in Table 3. *T. schoenleinii* strains showed increased sensitivity against fluconazole compared to the two genotypes of *T. quinckeanum*. The itraconazole behavior of *T. schoenleinii* resembled more the sensitive type of *T. quinckeanum* IHEM strains. The highest sensitivity to both azoles was found for *T. mentagrophytes* strain ATCC 46950. However, the agar-based method did not reach the accuracy of the MLN assay and produced more of an estimate.

### 3.2. Gene Analyses for Putative Association with Resistance Phenotypes

Azoles interact with sterol 14-α demethylases (Erg11); therefore, the two copies of *Erg11*, labeled A and B, were candidate genes to be analyzed. Point mutations in the gene for squalene epoxidase *Erg1* of *T. indotineae* were shown to be associated with an increase in azole resistance [23,31]. When comparing the sequences of *Erg11B* and *Erg1* of the *T. quinckeanum* ITS genotypes including *T. schoenleinii*, no nucleotide exchanges within the aligned fragments were observed (Table 4). The sequence of the neotype strain IHEM 13697 [8] was used as reference sequence. DNA sequence gaps were not counted in statistical analysis (Table 4). The results for *Erg11A* showed a higher level of nucleotide exchanges between the two ITS genotypes of *T. quinckeanum* compared to *T. schoenleinii* and the *T. quinckeanum* IHEM strains (Table 4).

Interestingly, Erg11A protein sequence alignments differ from the results obtained at the nucleotide level. Only two amino acid positions differed in the Erg11A protein alignments of both *T. quinckeanum* genotypes, whereas four amino acid exchanges were found in the protein sequences of IHEM *T. quinckeanum* and *T. schoenleinii*. A comparison of IHEM *T. quinckeanum* sequences with *T. simii* also revealed four amino acid exchanges. The Phe61Ser mutation is unique for IHEM *T. quinckeanum* strains compared to UKJ *T. quinckeanum*, *T. schoenleinii* and *T. simii*, where a Phe codon was found in this position. The UKJ *T. quinckeanum* Ala254Ser mutation differs from the Ala codon found in IHEM *T. quinckeanum*, *T. schoenleinii* and *T. simii* at this protein sequence position. Specific to *T. schoenleinii* were exchanges at Asp69Asn, Pro215His and Met306Ile. *T. simii* exhibited specific exchanges at Val316Ala, Val389Ile and Pro420Leu of the Erg11A protein sequence. Amino acids positions were deduced from the complete gene sequences decoded from the *T. schoenleinii* genome [36]. Interestingly, the *T. schoenleinii*-specific Pro215His mutation is part of the F-F’ motif, which is necessary for the function of Erg11 proteins [37].

### 3.3. T. quinckeanum and T. schoenleinii Represent Opposite Mating Types

Mating type genes were partially amplified as previously described [26] and sequenced. Results show that all *T. quinckeanum* strains belong to the minus mating type representing the *MAT1-1-1* gene [38] of the α-box transcription factor. Sequence information of the eight UKJ strains and the four IHEM strains of *T. quinckeanum* indicate the presence of the *MAT1-1-1* allele (Table 4). Interestingly, *T. schoenleinii* strains represent the opposite mating type, and the *Mat1-2-1* allele of the HMG-box transcription factor [34] was detected (Table 4). Other *T. quinckeanum* UKJ strains were analyzed using PCR and agarose gel electrophoresis (Figure S2). Thirty-six additionally analyzed *T. quinckeanum* UKJ strains were also of the minus mating type; so far, no isolate was identified that belongs to the opposite mating type. *T. mentagrophytes* strain ATCC 46950 also features the minus mating type (Table 4). Nevertheless, 39 nucleotide exchanges were counted when compared to *T. quinckeanum* IHEM 13697 sequences (Table 4).

## 4. Discussion

Both *T. quinckeanum* genotypes differ in the azole resistance pattern, in particular for itraconazole. It is likely that selection for azole-resistant strains began decades ago. Nowadays, sensitive strains of the IHEM genotype seem to have disappeared from the fungal population. Azoles belonging to the group of imidazole and triazole, for example, fluconazole and tebuconazole, have been widely used in agriculture for crop protection [39]. The risk of cross-resistance developing in non-phytopathogenic fungi has already been described for *Aspergillus fumigatus* [40], but also for other putative human fungal pathogens [39]. In accordance, the global emergence of antifungal resistance has been identified as an urgent public health threat, which requires a One Health approach [41,42]. Azole exposure of field mice in agriculture may drive selection pressure for azole resistance of zoophilic fungi of animals living in agricultural environments. There is a direct link between the environment and wellbeing [42], and increased awareness is needed for the crossing points of agriculture, animals and humans affecting our health [41]. There is also great danger when such a zoophilic *Trichophyton* species changes its host preference, as has happened in the past and as was the case, for example, with *T. indotineae*.

Putative genes involved in azole resistance only show diversification of *Erg11A*, which encodes one sterol 14α-demethylase. Other genes like *Erg11B* and *Erg1* feature no differences in nucleotide sequences when comparing sensitive and resistant *T. quinckeanum* strains.

The high number of silent point mutations found in *Erg11A* of the new *T. quinckeanum* genotype strains do not support the hypothesis of sole clonal reproduction. Since the *T. quinckeanum* strains of both genotypes belong to the same mating type, the question arises as to which species could serve as a mating partner. The imbalance in the mating type distribution was demonstrated in this study for both the *T. quinckeanum* strains of the IHEM genotype [14] and for the new *T. quinckeanum* genotype. Nevertheless, *T. quinckeanum* inter-species mating [14] was observed with ascopore isolates of *T. benhamiae var. benhamiae* (according to the new classification system [17]) and with *T. simii* [14]. Interestingly, meiotic F1 progeny of *T. quinckeanum* with *T. benhamiae var. benhamiae* behaves in mating type distribution as expected from Mendelian rules [14]. Mating of *T. quinckeanum* and *T. simii,* in contrast, produces unusual F1 recombinants in which only one mating type is dominant, leading to an imbalance in the mating type distribution [14]. A similar phenomenon of unusual recombinants leading to an unbalanced distribution of mating types was observed in crosses between *T. europaeum* (formerly white subtype of *T. benhamiae*) and *T. benhamiae var. benhamiae* [26].

Phylogenetic analyses of several genes showed that *T. quinckeanum* and *T. simii* are closely related, while the *T. benhamiae* complex represents a somewhat more distant relation [4,8]. They also showed the close relationship of *T. schoenleinii* and *T. quinckeanum*, which could also be considered as conspecifics. Both species belong to opposite mating types, and perhaps *T. schoenleinii* appeared as a mating partner of *T. quinckeanum* in the past. However, for the new *T. quinckeanum* strains, both *T. schoenleinii* and *T. simii* cannot be the donor of the *Erg11A* gene. So far, no genome information is available for the unknown donor species. The environmental conditions of the unknown donor species must be very similar to those of *T. quinckeanum*, which explains the high similarity of the Erg11A protein sequences, while many silent point mutations were detected on the nucleotide level. The phylogenetic distance of *T. quinckeanum* and the donor is comparable to that of *T. simii*.

Metin and Heitman [24] postulated that anthropophilic *Trichophyton* strains consist of one mating type, while zoophilic strains consist of two mating types. Interestingly, even when both mating types belong to closely related species, like *T. europaeum* and *T. japonicum* (previously described as white subtypes of *T. benhamiae*), no recombinants are detected from natural obtained isolates [17]. Nevertheless, both species are able to produce viable ascospores [18,26]. The phenomenon of recombinants not following Mendelian rules explains the mating type imbalance and preferential parental genotypes of meiotic ascospores. In a postulated mating system in which recombinants are nearly suppressed, parental genotypes recreated by meiosis and clonal reproduction is mimicked. Meiosis is a powerful tool to remove defective genes from the genome and is important for successful adaptation to new environmental conditions [43,44,45]. Suppression of recombinants from closely related species enhances speciation and helps adaptation when subtypes prefer different host species. The ability to also mate with more distant related species that produces F1 progeny according to Mendelian rules could be understand as a putative exit strategy to avoid extinction if host population decreases.

In conclusion, our study highlights the importance of monitoring trends in dermatophyte species and emergence of resistance. New genotypes of dermatophytes may evolve by mating and due to selection pressure. A limitation of the study is the fact that it is single-centered, and only *T. quinckeanum* strains from Thuringia are examined. Hence, the occurrence of this new genotype could be interpreted as a local phenomenon. However, recent reports from other regions [9,10,11] clearly indicate a wider distribution and a general trend for the selection towards this new, azole-resistant genotype of *T. quinckeanum*. Hence, treatment of infections with these strains will likely present a challenge to clinicians for managing dermatophytosis in the future, which is especially troublesome in the light of the development of severe Tinea capitis infections (Kerion celsi) in children. In accordance with the call made by Fisher et al. to establish a global network including policymakers, funders, researchers, antifungal producers and product users [42], the next step for elucidating the distribution of the new genotype of *T. quinckeanum* and its antifungal resistance could be implementation of a national study.

## Figures and Tables

**Table 1:** Source information for strains used in MLN or plate assays to determine fungal resistance.

Species	Strain ^1^	Synonyms ^1^	Collected at Year	Country	Host	Age, Sex	Diagnosis
*T. quinckeanum*	UKJ 1505/12		2012	Germany	Human	6 y, female	Tinea corporis
	UKJ 621/13		2013	Germany	Human	male	Tinea corporis
	UKJ 1547/14		2014	Germany	Human	7 y, female	Tinea capitis
	UKJ 1905/19		2019	Germany	Human	25 y, female	Tinea manum
	UKJ 1953/19		2019	Germany	Cat		Infected hairs
	UKJ 1254/20		2020	Germany	Cat		Infected hairs
	UKJ 1506/20		2020	Germany	Human	69 y, female	Tinea corporis
	UKJ 1891/20		2020	Germany	Human	21 y, female	Tinea corporis
	IHEM 13570	CDC X395	Before 1968 ^2^	USA	Dog		Favus ^4^
	IHEM 13572		1964	Australia	Rodent		Favus ^4^
	IHEM 13697	CDC X393	Before 1968 ^2^	USA	Mouse		Favus ^4^
	IHEM 26522	CBS 318.56	1955	Netherlands	Human		Deep trichophytosis ^4^
*T. schoenleinii*	IHEM 13515		1966	Morocco	Human		Favus ^4^
	UKJ 1317/12		ap. 1972				
*T. mentagrophytes*	ATCC 46950		Before 1980 ^3^	Iraq	Human		Infected epilated hairs

^1^ The prefix UKJ indicates that strains belong to strain collection of Jena University Hospital. IHEM-designated strains were obtained from Belgian Coordinated Collection of Microorganisms BCCM (Belgium), and ATCC-named strains were obtained from American Type Culture Collection (USA). The abbreviation CBS stands for CBS-KNAW Culture Collection, Westerdijk Fungal Biodiversity Institute (Utrecht, The Netherlands), and CDC refers to a former numbering by the Centers for Disease Control and Prevention (Atlanta, GA, USA) as cited here [4,13]. ^2^ The strains’ descriptions were first published [13] with reference to them being a gift from F. Blank to the authors without a specific collection date. ^3^ The capability of infected epilated hairs leading to fungal cultures were studied over a time range of four years by the author before submission to the strain collection [27]. ^4^ Information as obtained from IHEM or CBS strain descriptions [28,29].

**Table 2:** MIC_90_ values of *T. quinckeanum* strains.

Strain	Itr	Vor	Ser	Clt	Ter	Amo	Nys	Cic
UKJ 1505/12	0.45 ± 0.13	0.71 ± 0.04	2.78 ± 0.66	0.42 ± 0.02	0.0038 ± 0.0006	0.021 ± 0.004	16.4 ± 1.9	7.5 ± 1.0
UKJ 621/13	0.12 ± 0.001	0.70 ± 0.10	3.81 ± 0.11	0.39 ± 0.01	0.0030 ± 0.0005	0.053 ± 0.0004	15.6 ± 0.5	7.4 ± 0.8
UKJ 1547/14	0.29 ± 0.11	0.59 ± 0.06	1.88 ± 0.22	0.25 ± 0.02	0.0030 ± 0.0002	0.026 ± 0.001	11.0 ± 1.5	6.4 ± 0.9
UKJ 1905/19	0.11 ± 0.004	0.55 ± 0.04	2.03 ± 0.01	0.20 ± 0.004	0.0030 ± 0.0002	0.027 ± 0.001	10.0 ± 0.2	8.7 ± 2.4
UKJ 1953/19	0.25 ± 0.13	0.99 ± 0.28	1.60 ± 0.23	0.22 ± 0.06	0.0030 ± 0.0005	0.019 ± 0.001	18.9 ± 2.6	6.4 ± 0.3
UKJ 1254/20	0.11 ± 0.02	0.88 ± 0.01	1.48 ± 0.34	0.18 ± 0.06	0.0026 ± 0.0002	0.018 ± 0.0004	19.4 ± 0.9	7.5 ± 1.5
UKJ 1506/20	0.34 ± 0.061	1.03 ± 0.15	1.49 ± 0.13	0.25 ± 0.03	0.0029 ± 0.0004	0.020 ± 0.0001	10.5 ± 0.3	7.0 ± 0.9
UKJ 1891/20	0.091 ± 0.02	0.47 ± 0.06	1.96 ± 0.44	0.22 ± 0.01	0.0028 ± 0.0002	0.034 ± 0.001	9.9 ± 0.6	6.7 ± 0.9
IHEM 13570	0.0033 ± 0.0004	0.11 ± 0.009	0.81 ± 0.16	0.06 ± 0.02	0.0026 ± 0.0003	0.014 ± 0.0001	5.4 ± 0.4	4.9 ± 1.8
IHEM 13572	0.070 ± 0.007	0.53 ± 0.10	1.88 ± 0.17	0.26 ± 0.02	0.0036 ± 0.0007	0.024 ± 0.004	10.1 ± 3.1	7.9 ± 0.9
IHEM 13697	0.0030 ± 0.00007	0.17 ± 0.003	0.67 ± 0.19	0.08 ± 0.01	0.0016 ± 0.0001	0.015 ± 0.001	4.1 ± 0.6	12.5 ± 0.4
IHEM 26522	0.019 ± 0.001	0.62 ± 0.07	1.66 ± 0.13	0.22 ± 0.04	0.0020 ± 0.0002	0.025 ± 0.002	10.8 ± 0.7	7.1 ± 0.3

The following antifungal compounds were analyzed by microplate laser nephelomtric (MLN) assay: itraconazole (Itr), voriconazole (Vor), sertaconazolenitrate (Ser), clotrimazole (Clt), terbinafin (Ter), amorolfin (Amo), nystatin (Nys) and clicopirox olamine (Cic). All UKJ strains represent the new genotype, whereas all IHEM strains represent the old genotype. Values denote average of antifungal concentration in µg/mL and standard deviation.

**Table 3:** Growth on SDA plates doped with increasing concentrations of fluconazole (Flu) or itraconazole (Itr).

Species	Strain *	Flu 0.4 µg/mL	Flu 4 µg/mL	Flu 40 µg/mL	Itr 0.005 µg/mL	Itr 0.05 µg/mL	Itr 0.5 µg/mL
*T. quinckeanum*	UKJ 1505/12	n.t.	+	+	n.t.	+	+
	UKJ 621/13	n.t.	+	+	n.t.	+	+
	UKJ 1547/14	n.t.	+	+	n.t.	+	+
	UKJ 1905/19	n.t.	+	+	n.t.	+	+
	UKJ 1953/19	n.t.	+	+	n.t.	+	+
	UKJ 1254/20	n.t.	+	+	n.t.	+	+
	UKJ 1506/20	n.t.	+	+	n.t.	+	+
	UKJ 1891/20	n.t.	+	+	n.t.	+	+
	IHEM 13570	n.t.	+	+	n.t.	+/−	−
	IHEM 13572	n.t.	+	+	n.t.	+/−	−
	IHEM 13697	n.t.	+	+	n.t.	+/−	−
	IHEM 26522	n.t.	+	+	n.t.	+/−	−
*T. schoenleinii*	UKJ 1317/12	+	+	−	+	−	−
	IHEM 13515	+	+	−	+	−	−
*T. mentagrophytes*	ATCC 46950	+	−	−	−	−	−

* If the mycelium was able to grow into the agar plate, the result was scored as positive (+), and if mycelium remained confided to the transferred small region, the growth was scored as negative (−). When small amounts of mycelium showed limited growth on the azole-containing agar, the reaction was labeled intermediate (+/−). Concentrations not tested were marked as n.t. (not tested).

**Table 4:** Multiple gene fragment alignments of *T. quinckeanum* and *T. schoenleinii* strains.

Species	Strain Alignment in bp *	*Erg11A* 1509 bp	*Erg11B* 1722 bp	*Erg1* 1420 bp	*Mat1-1-1* 571 bp	*Mat1-2-1* 1121 bp
*T. quinckeanum*	UKJ 1505/12	15	0	0	1	absent
	UKJ 621/13	15	0	0	1	absent
	UKJ 1547/14	15	0	0	1	absent
	UKJ 1905/19	15	0	0	1	absent
	UKJ 1953/19	15	0	0	1	absent
	UKJ 1254/20	15	0	0	1	absent
	UKJ 1506/20	15	0	0	1	absent
	UKJ 1891/20	15	0	0	1	absent
	IHEM 13570	0	0	0	1	absent
	IHEM 13572	1	0	0	1	absent
	IHEM 13697	0	0	0	0	absent
	IHEM 26522	0	0	0	1	absent
*T. schoenleinii*	UKJ 1317/12	8	0	0	absent	0
	HEM 13515	9	0	0	absent	0
	CMCC (F)T2s	8	0	0	absent	0
*T. simii*	HEM 4420	20	30	22	7	absent
*T. mentagrophytes*	ATCC 46950	75	66	65	39	absent

* IHEM neotype strain 13697 was selected as the reference sequence. Numbers represent amounts of nucleotide exchanges found in alignments of the different strains compared to the reference sequence. IHEM strain 13515 chosen as reference for the plus mating type specific fragment *Mat1-2-1*. Genomic data from *T. schoenleinii* CMCC (F)T2s [36] confirm species identification of IHEM 13570 and UKJ 1317/12. Data from *T. simii* IHEM 4420 were obtained from a bioproject of GenBank Acc. No. PRJNA656715.

## Data Availability

Sequence data were submitted to GenBank and are publicly available. Detailed information is presented in Table S2.

## Supplementary Materials

The following supporting information can be downloaded at: https://www.mdpi.com/article/10.3390/jof9101006/s1, Figure S1: Phylogenetic tree of ITS sequences of *T. quinckeanum* and related species using the Neighbor Joining method; Figure S2: Mating type analysis of *T. quinckeanum* strains; Table S1: Adjusted primer list for amplification of *T. quinckeanum* fragments; Table S2: GenBank Acc. No. of sequenced DNA fragments. References [4,6,26,35,46,47,48,49] are cited in the supplementary materials.
