# Supplementary material for: A New Genotype of Trichophyton quinckeanum with Point Mutations in Erg11A Encoding Sterol 14-α Demethylase Exhibits Increased Itraconazole Resistance
Source: J Fungi (Basel). 2023 Oct 12;9(10):1006. doi: 10.3390/jof9101006 (PMC10607888; doi:10.3390/jof9101006)
Supplement: Supplementary file 1 [file jof-09-01006-s001.zip › jof-2597781-supplementary.pdf]

Journal of *Fungi*

MDPI

A new genotype of *Trichophyton quinckeanum* with point mutations in *Erg11A* encoding sterol 14-α demethylase exhibits increased itraconazole resistance

Paula Winter, Anke Burmester, Jörg Tittelbach and Cornelia Wiegand

Supplementary Materials

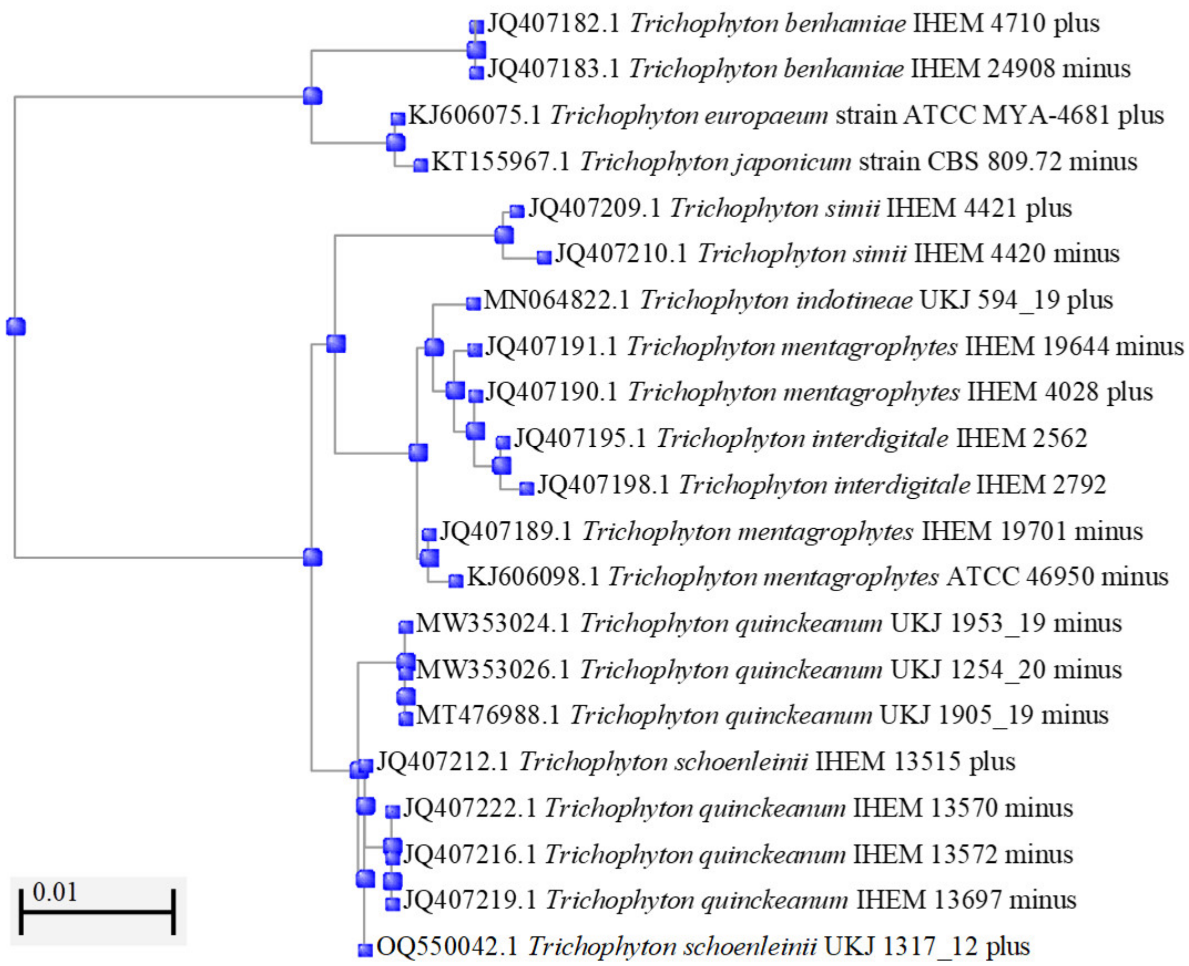

Figure S1. Phylogenetic tree of ITS sequences of *T. quinckeanum* and related species using Neighbor Joining method [35]. Accession No., species name and strain designation were shown. Mating type MAT1-2-1 were described as plus and the opposite mating type MAT1-1-1 as minus. Program Unipro UGENE was used for assembling genes and alignments of fragments of equal lengths [46]. Phylogenetic tree was obtained by use of the online tools of NCBI (National Center for Biotechnology Information, USA)[47]. Standard conditions were used for building the phylogenetic tree with a setting of 0.75 for maximal sequence difference.

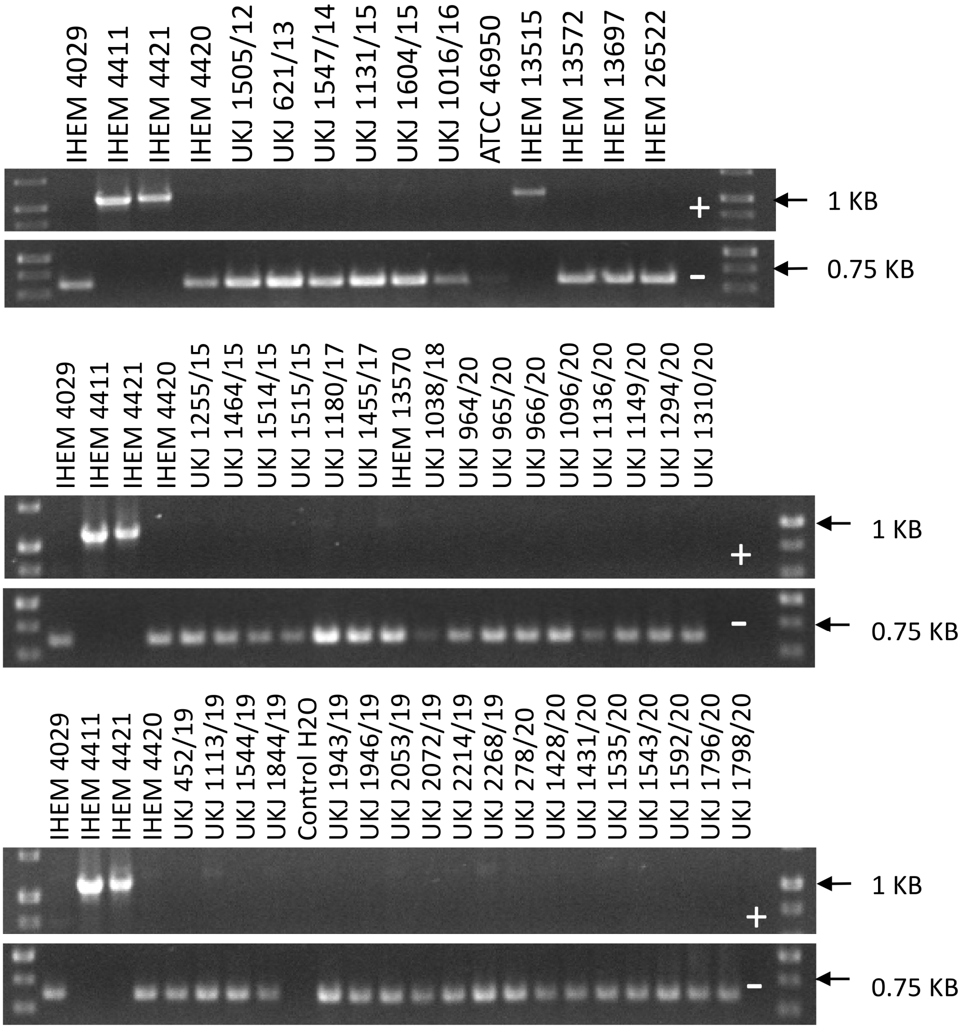

Figure S2. Mating type analysis of *T. quinckeanum* strains. The genes of mating type MAT1-1-1 (α-box transcription factor like), the minus mating type, or MAT1-2-1 (HMG-box transcription factor like), the plus mating type, were partially amplified and analyzed by agarose gel electrophoresis as described previously [26]. As controls for minus mating types served DNA amplified from *T. mentagrophytes* IHEM 4029 strain and *T. simii* strain 4420. For controls of opposite mating types, PCR fragments from *T. mentagrophytes* strain IHEM 4411 and *T. simii* strain IHEM 4421 were analyzed.

Table S1. : **Adjusted primer list for amplification of *T. quinckeanum* fragments.**

Gene	Primer name	Sequence 5′3′
HMG (MAT1-2-1)	TqHMG_F1	ACCATGCCGATGCCAGCATCTG
	TqHMG_R1	CGAAATCAACATCAGTTGTCC
Erg1	TqErg1_F5	TGGGGGCTGGAGCTTATAGATG
	TqErg1_R5	CAAATCACCCAGAACAACATGG
Erg11A	TqErg11A_F1	GGATGTGAATGCAGAAGAGG
	TqErg11A_R1	TAGACGAGTTGAGGCGGAGG
	TqErg11A_F2	CTCAATGTCGTCTATCAGCTGC
	TqErg11A_IR2	TATGGTTGGGCTTGGCTCCTTC
	TqErg11A_UR2	TATGATTGGGCTTGGATCCTTC
	TqErg11A_F3	GCACATATGATGATCACCCTCC
	TsErg11A_R3	ACTGTTCAATTCAATGTGAC
Erg11B	TqErg11BF2	GCCCACATGATGATCGCTCTTC

Table S2. : **GenBank Acc. No. of sequenced DNA fragments.**

Species	Strain GenBank	*Erg11A* Acc. No.	*Erg11B* Acc. No.	*Erg1* Acc. No.	*Mat1-1-1* Acc. No.	*Mat1-2-1* Acc. No.	*ITS* Acc. No.*
T. quinckeanum	UKJ1505/12	OQ536520	OQ536535	OQ536505	OQ536550	absent	OQ550042
	UKJ621/13	OQ536521	OQ536536	OQ536506	OQ536551	absent	OQ550043
	UKJ1547/14	OQ536522	OQ536537	OQ536507	OQ536552	absent	OQ550044
	UKJ1905/19	OQ536523	OQ536538	OQ536508	OQ536553	absent	MT476988
	UKJ1953/19	OQ536524	OQ536539	OQ536509	OQ536554	absent	MW353024
	UKJ1254/20	OQ536525	OQ536540	OQ536510	OQ536555	absent	MW353026
	UKJ1506/20	OQ536526	OQ536541	OQ536511	OQ536556	absent	MW353028
	UKJ1891/20	OQ536527	OQ536542	OQ536512	OQ536557	absent	OQ550045
	IHEM13570	OQ536528	OQ536543	OQ536513	OQ536558	absent	JQ407222
	IHEM13572	OQ536529	OQ536544	OQ536514	OQ536559	absent	JQ407216
	IHEM13697	OQ536530	OQ536545	OQ536515	OQ536560	absent	JQ407219
	IHEM26522	OQ536531	OQ536546	OQ536516	OQ536561	absent	MK298974
T. schoenleinii	UKJ1317/12	OQ536532	OQ536547	OQ536517	absent	OQ536562	OQ550046
	HEM13515	OQ536533	OQ536548	OQ536518	absent	OQ536563	JQ407212
T. mentagrophytes	ATCC46950	OQ536534	OQ536549	OQ536519	OQ536564	absent	KJ606098

*Acc. No. starting with MT or MW were as published [6], JQ No. in [4], MK No. in [48], KJ No. in [49].
